# *STAC3* gene congenital myopathy and malignant hyperthermia: a crossroads between neurology and anesthesia

**DOI:** 10.1055/s-0045-1806734

**Published:** 2025-04-22

**Authors:** Mary Santos Silva, Ricardo Nakamura, Marcia Rosana Arjona, Thue Peres Colferai Del Monaco, Mauricio Luiz Malito, Taís Oliveira Sampaio, Samantha Lopes de Oliveira, Juliana Silva de Almeida Magalhães, Marcela Camara Machado-Costa, Helga Cristina Almeida Silva

**Affiliations:** 1 Universidade Federal de São Paulo, Escola Paulista de Medicina, Departamento de Anestesiologia, Dor e Medicina Intensiva, São Paulo SP, Brazil.; 2 Hospital e Maternidade Sepaco, São Paulo SP, Brazil.; 3 Associação de Assistência à Criança Deficiente, São Paulo SP, Brazil.; 4 Hospital Martagão Gesteira, Salvador BA, Brazil.; 5 Escola Bahiana de Medicina e Saúde Pública, Salvador BA, Brazil.

**Keywords:** Anesthesia, Malignant Hyperthermia, Muscular Diseases

## Abstract

*STAC3* gene congenital myopathy and malignant hyperthermia (MH) represent an important crossroads between neurology and anesthesia, where the prompt recognition of the clinical characteristics, and the collaboration between neurologists and anesthesiologists, are essential to early diagnosis and prevention of adverse critical events. This gene is associated with a congenital myopathy first reported as Native American myopathy (NAM), a rare condition characterized by dysmorphisms, contractures, muscular complaints, and scoliosis. As a rare pharmacogenetic hypermetabolic disease, MH is triggered by halogenated agents and/or succinylcholine, linked to variants in the *RYR1,* *CACNA1S,* or *STAC3* genes. Our objective was to analyze the characteristics of a Brazilian case series of *STAC3* gene myopathy associated with MH and to review previous reports. We report three MH crises, in two boys and one girl, 2 to 15 years old. All of them received halogenated agents and one additionally received succinylcholine. Two patients presented two to four previous uneventful general anesthesia. The MH crises in this series of patients with *STAC3* gene mutations demonstrated variable clinical characteristics (expressivity) and occurrence (penetrance). Neuromuscular patients with findings suggestive of *STAC3* myopathy should increase diagnostic suspicion regarding the risk of MH. Conversely, the careful evaluation of the anesthetic antecedents of neuromuscular patients can help to restrict the candidate genes. Additionally, Brazilian neurologists can notify neurological patients with antecedents of adverse events during anesthesia to the Brazilian Registry of Neurological Diseases (Registro Brasileiro de Doenças Neurológicas, REDONE, in Portuguese).

## INTRODUCTION

The *STAC3* gene is associated with congenital myopathy and malignant hyperthermia (MH), representing an important crossroads between neurology and anesthesia, where the prompt recognition of the clinical characteristics and the collaboration between neurologists and anesthesiologists are essential to early diagnosis and prevention of adverse critical events.

Native American myopathy (NAM) is a neuromuscular disease characterized by congenital myopathy with dysmorphisms and susceptibility to MH. It was described in 1987 in an Indigenous child belonging to the Lumbee tribe in North Carolina, United States. 1 2 Other reports in the same tribe followed, and NAM was associated with homozygous pathogenic variants in the *STAC3* gene (c.851G > C, p.Trp284Ser), which encodes a protein associated with ryanodine/dihydropyridine receptors, involved in the process of muscle excitation-contraction coupling. 3 Initially, NAM was thought to be exclusive to the Lumbee group, but patients with similar clinical characteristics and homozygous/compound heterozygous variants in the *STAC3* gene began to be described worldwide (in Africa, Puerto Rico, the Middle East, Turkey, the Caribbean, the Comoro Islands). 4 5 A review of the origins of the Lumbee tribe indicated that there was an admixture of Cheraw Indian, African American, and English ancestry. 5 These data led to the proposal of revising the name from NAM to congenital myopathy linked to the *STAC3* gene. 5

Regarding MH, it is a potentially fatal, autosomal, dominant, pharmacogenetic disease following the exposure of genetically susceptible individuals to halogenated agents/succinylcholine and characterized by hypermetabolism, with tachycardia, hypercapnia, hyperthermia, muscle rigidity, increased oxygen consumption, and lactic acidosis, followed by rhabdomyolysis, disseminated intravascular coagulation, and multiple organ failure. 1 2 Treatment is based on interrupting the triggering agents, administering the antidote dantrolene, and controlling complications. 2 The standard diagnostic test is the in vitro muscle contracture test (IVCT) in response to halothane and caffeine, also known as the caffeine-halothane contracture test. 1 Furthermore, MH presents clinical/genetic variability, so susceptible individuals may be asymptomatic or exhibit muscle hypertrophy/atrophy. While 23 to 86% of families have variants in the *RYR1* gene, there are rare cases which present variants in the *CACNA1S* or *STAC3* genes. 1

Despite the extensive clinical characterization of NAM, there are no systematic studies of the clinical/laboratory characteristics of the patients who presented MH crises. Therefore, the objective of this article was to analyze the characteristics of a Brazilian case series of *STAC3* gene myopathy associated with MH and to review previous reports.

## REPORT

The present study was approved by the Ethics in Research Committee of the Universidade Federal de São Paulo, under number 0979/2017, and a free and informed written consent form was obtained from all the families. We identified three patients with variants in the *STAC3* gene and history of MH crisis. Their ages ranged from 2 to 15 years, with two boys and one girl. All of them were Afro-Brazilian, with the same homozygous variant in the *STAC3* gene, despite no reports of consanguinity in the families ( Table 1 ). None of the three patients reported Native American ancestry, assessed by the absence of known indigenous ancestors.

**Table 1:** Occurrence of MH crisis related to the *STAC3* gene: case series

Patient	Age; sex; ethnicity; *STAC3* variant	Clinical history and findings	Surgery	Anesthetics	MH crisis	Evolution clinical grade scale
1	2 y; M; AB; homozygous c.851G > C p.(Trp284Ser)	2 previous uneventful inhalational GA: 1 gastrostomy, 1 postectomy/orchidopexy. Motor delay (meconium aspiration?), scoliosis (Cob 70°), cryptorchidism, clubfoot, flexion contractures of the 3rd/4th/5th fingers. Elongated facies, bilateral ptosis, high arched palate, facial diparesis, muscular hypotrophy, cervical and proximal (3 + ) and distal (4) paresis, hypotonia, global areflexia; no consanguinity. Mother: miscarriage at 12 weeks; father: clubfoot	Clubfoot correction	Sevoflurane	1st signal at 10 min: hypercarbia; Hypercarbia (ETCO2 133 mmHg), tachycardia (152–179 bpm), hyperthermia (38.6°C), acidosis (venous pH 7.2).	Improvement after sevoflurane suspension; CGS **:** 33
2	12 y; M; AB; homozygous c.851G > C p.(Trp284Ser)	4 previous uneventful inhalational GA: orchidopexy, gastrostomy/tracheostomy closure, foot fracture correction, spinal instrumentation without fusion (growth system). Motor delay, progressive scoliosis, cryptorchidism, muscle weakness; no consanguinity. Sister: cleft lip and dysphagia	Distraction of the spinal growth system	Sevoflurane	1st sign at 15 min: muscle rigidity (masseter, limbs, chest, neck); Hypercarbia (ETCO2 120 mmHg), tachycardia (170 bpm), hyperthermia (39.9°C), acidosis (pH 6.92, BE-5.7), hypotension (40 × 10 mmHg).	Improvement after sevoflurane suspension, hyperventilation with 100% oxygen, IV dantrolene (2.5 and 2 mg.kg− ^1^ ), adrenaline; CGS: 63
3	15 y; F; AB; homozygous c.851G > C p.(Trp284Ser)	No consanguinity; motor delay, progressive scoliosis (Cob 53°), joint hypermobility, contractures in fingers and hands; short stature, elongated facies, bilateral ptosis, high palate, facial diparesis, muscular hypotrophy, grade 4 limb paresis, hypotonia, global areflexia	Spinal arthrodesis	Succinylcholine; sevoflurane	1st sign at 15 min: masseter rigidity; Global muscle rigidity, hypercarbia (ETCO2 61 mmHg), hyperthermia (37.8°C), acidosis (pH 7.28), hypotension (80 × 50 mmHg), rhabdomyolysis (CK 1152, normal up to 140 IU/l).	Improvement after sevoflurane suspension, hyperventilation, IV dantrolene (bolus 2.5 mg.kg− ^1^ , maintenance 0.25 mg.kg− ^1^ .h− ^1^ ); CGS: 50

Abbreviations: AB, Afro-Brazilian; bpm, beats per minute; CGS, clinical grade scale; CK, creatine kinase; min, minutes; ETCO2, End-tidal carbon dioxide; F, female; GA, general anesthesia; M, male, MH, malignant hyperthermia; y, years.

The two boys had cryptorchidism and previous inhalation anesthesia with halogenated agents, without complications. The sister of patient number two had a cleft lip. All three patients presented with motor delay, scoliosis, and muscle weakness. Additionally, patients one and three had contractures and dysmorphic features described in the NAM (elongated facies and eyelid ptosis).

All three MH crises were triggered by sevoflurane, with concomitant use of succinylcholine in one patient, and had a rapid onset, between 10 and 15 minutes after the start of the halogenated drug, with increased levels of exhaled carbon dioxide (hypercarbia) and muscle rigidity. Notably, patients one and two developed intense hypercarbia (120–133 mmHg, normal range: 35–45 mmHg).

A specific clinical grading score was used for estimating the probability of each crisis being MH. This score evaluates the presence of rigidity, evidence of rhabdomyolysis, acidosis (respiratory, metabolic), hyperthermia, cardiac problems, improvement after treatment with dantrolene, and personal/family MH history. This score can have minimum/maximum absolute values from 0 to 88, corresponding to probabilities from almost never (0) to almost certain (≥ 50). In the three patients of this report, this score ranged from 33 to 63 points, corresponding to a probability of MH crisis from somewhat greater than likely to almost certain. 6

All patients had good clinical outcomes and survived the MH crisis. One of them improved with the halogenated drug suspension, while the other two also received the specific MH antidote dantrolene. None performed the IVCT, but a genetic test detected the same homozygous variant in the *STAC3* gene.

## REVIEW

The NAM phenotype ranges from severe congenital disease to slowly progressive congenital myopathy, as exemplified by the patients in the present report. The congenital features of NAM patients, some of which may become more pronounced with age, may include short stature, elongated face with inability to elevate the corners of the mouth, ears with posterior rotation and low implantation (below eye level), eyelid ptosis, descending palpebral fissure, telecanthus, micrognathia, high arched palate, cleft palate, bifid uvula, cryptorchidism, joint hyperextensibility, hearing deficits, facial hemangiomas, clubfoot, and multiple joint contractures (arthrogryposis). 2 3 4 5 Muscle weakness has been described in the upper and lower limbs, as well as in the facial, paravertebral (leading to kyphosis and/or scoliosis), respiratory (restrictive respiratory failure), and oropharyngeal (dysphagia, deficit in weight gain) muscles. 2 4 5 This weakness causes hypotonia and delays the acquisition of motor milestones, such as sitting, walking, running, and jumping. Basal creatine kinase (CK) is often within normal limits.

We found five more reports of MH crises in patients belonging to the Lumbee group (n = 1) or with variants in the *STAC3* gene (n = 4), as shown in Table 2 . 2 7 8 9 Their ages ranged from 3 months to 15-years-old, with two males and three females. Three patients were homozygous for the same *STAC3* gene variant found in our patients, while one was compound heterozygous. Furthermore, two of the five patients had one to five previous general anesthesia without complications. All five MH crises were associated with halogenated agents. Notably, in two of the five patients, muscle rigidity was the only sign noted. Furthermore, three of the five patients did not develop signs of rhabdomyolysis.

**Table 2:** Occurrence of MH crisis related to the *STAC3* gene previously published

Author, year	Age; sex; *STAC3* variant	Clinical history and findings	Surgery	Anesthetics	MH crisis	Evolution Clinical grade scale
Bailey and Bloch, 1987 2	3 m; F; (first description: Lumbee group)	One previous uneventful GA, micrognathia, cleft palate, talipes equinus, arthrogryposis, periodic apnea, dysphagia, gastroesophageal reflux, T12-L1 syrinx	Gastrostomy	Halothane, nitrous oxide/oxygen, atracurium	5 min: hypercapnia (ETCO2 70 mmHg), tachycardia (205 bpm), muscle rigidity, hyperthermia (38°C), ↑ CK (1,470, upper limit: 160 IU/l)	Dantrolene with improvement; CGS: 53
Telegrafi et al., 2017 7	15 y; F; c.851G > C; p.Trp284Ser (homozygous)	Hypotonia, cleft palate, ptosis, hearing loss, facial diplegia, kyphoscoliosis	Spine surgery	Inhaled agents	Body stiffening	Treatment for MH; Hypotension after blood loss; neurologic injury; CGS: 15
Zaharieva et al., 2018 8	6 m; M; c.851G *>* C, p.(Trp284Ser)/c.997−1G *>* T	Five previous uneventful GAs, hypotonia, bifid uvula, ptosis, hearing loss, myopathic face, multiple contractures (elbow, fingers, ankles), global muscle weakness, calves atrophy, respiratory/feeding impairment, kyphoscoliosis, cryptorchidism	Intravenous access	Sevoflurane, nitrous oxide/oxygen	45 min: ↑ upper / lower limb muscle tone, hypercapnia, tachycardia (208 bpm), hyperthermia (39.6°C)	Dantrolene, with total improvement; No rhabdomyolysis or hyperkalemia; CGS: 53
Gromand et al., 2022 9	3,5 y and 9,5 y; M; c.851G *>* C; p.Trp284Ser (homozygous)	Neonatal hypotonia, cleft palate, ptosis, long face, downturned corners of the mouth, camptodactyly, Gowers sign, hyporeflexia	Tonsillectomy Tympanoplasty	Halogenated gas	Muscle rigidity, hypercapnia	Normal renal function; no rhabdomyolysis; CGS: 30
Gromand et al., 2022 9	1,5 y; F; c.851G *>* C; p.Trp284Ser (homozygous)	Neonatal hypotonia, cleft palate, ptosis, facial hypomimia, camptodactyly, hyporeflexia, hyperlaxity	Cleft palate correction	Halogenated gas	Muscle rigidity	No rhabdomyolysis; CGS: 15

Abbreviations: bpm, beats per minute; CGS, clinical grade scale; CK, creatine kinase; ETCO2, end-tidal carbon dioxide; F, female; GA, general anesthesia; m, months; M, male; MH, malignant hyperthermia; min, minutes; y, years.

In our three patients, the MH crisis had a rapid and fulminant onset, with a high clinical grading score, similar to that described in the reports of Bailey and Bloch 2 and Zaharieva et al. 8 Similar to these two reports, the history of previous inhalation anesthesia without complications reinforces that this information does not exclude the risk of MH in a family with *STAC3* gene variants. Stam et al. detected a 29% frequency of MH in a group of 14 patients with NAM, suggesting that the penetrance of mutations in the *STAC3* gene is incomplete regarding the ability to cause MH crisis. 10 Furthermore, there is variable expressivity, demonstrated by three reports of abortive or mild crises 7 9 and the variability of the clinical grading scale value. However, due to the retrospective analysis nature of the five previously published cases, there were not as many details as in our series of three cases, which could be associated with an underestimation of the clinical grading score.

Due to multiple abnormalities, these patients frequently undergo surgery, mainly for gastrostomy, correction of cleft palate or eyelid ptosis, kyphoscoliosis, clubfoot, or other contractures. In these cases, a high level of suspicion for the possibility of MH in patients with multiple dysmorphisms is imperative, with planning for evaluation with neuropediatrics, neurology, and genetics teams. Anesthesia without triggering agents (halogenated agents and succinylcholine) should be performed, in addition to cleaning the anesthetic machine for halogenated agents. Dantrolene and an intensive care unit must be available. All patients at MH risk must receive a report from their medical doctors warning about MH and indicating the necessary cautions. There is a Brazilian site (cedhima.unifesp.br) with additional information about MH and 24-hour phone service for advice, the Brazilian MH hotline (+55-11-55759873, +55-11-997457730).

The absence of consanguinity stands out among the three patients in this report, which suggests that mutations in the *STAC3* gene are not so rare. In a North American MH series, these variants were found in one of 57 patients. 1 Therefore, analysis of this gene should be included in investigations of patients who present with MH crisis so that families can be guided toward safe anesthetic measures. The variant present in our three families was the same as that reported in Lumbee patients with NAM. 3 In the Lumbee tribe, MH crises and NAM were described only in homozygous patients, so carriers of one *STAC3* gene variant appeared to be asymptomatic. 2 3 4 5 However, there is a report of an individual with a history of MH crisis, an abnormal muscle contracture test, and one variant of uncertain significance in the *STAC3* gene (c.560T > C, p.Met187Thr). 1

In this small sample of patients with mutations in the *STAC3* gene, MH crises demonstrated variable expressivity and penetrance, with characteristics similar to those MH crises described in patients with mutations in the *RYR1* gene. Our preliminary findings must be confirmed in larger series. Patients with dysmorphisms, contractures, muscular complaints, and scoliosis should increase diagnostic suspicion regarding the risk of MH. Conversely, the careful evaluation of the anesthetic antecedents of neuromuscular patients can help to restrict the candidate genes. Additionally, Brazilian neurologists can notify neurological patients with antecedents of adverse events during anesthesia to the Brazilian Registry of Neurological Diseases (Registro Brasileiro de Doenças Neurológicas, REDONE, in Portuguese), which aims to collect data on neurological diseases in Brazil, with the aim of contributing to public policies and academic research ( https://redone.med.br/estudo/hipertermia-maligna-estudo-multidisciplinar-brasileiro ).
